# Interstitial lung diseases with concomitant lung cancer: a data mining approach revealing a complex condition with gender- and immune-associated specific implications

**DOI:** 10.3389/fonc.2024.1488157

**Published:** 2024-12-17

**Authors:** Fabio Perrotta, Donato Lacedonia, Vito D’Agnano, Andrea Bianco, Giulia Scioscia, Pasquale Tondo, Maria Pia Foschino Barbaro, Francesca Mariani, Sara Lettieri, Lucia Del Frate, Silvia Mancinelli, Davide Piloni, Tiberio Oggionni, Chandra Bortolotto, Laura Carrozzi, Isa Cerveri, Angelo Guido Corsico, Giulia Maria Stella

**Affiliations:** ^1^ Department of Translational Medical Sciences, University of Campania “L. Vanvitelli”, Napoli, Italy; ^2^ Department of Medical and Surgical Sciences, University of Foggia, Foggia, Italy; ^3^ Department of Specialist Medicine, Institute of Respiratory Diseases, University-Hospital Polyclinic “Riuniti”, Foggia, Italy; ^4^ Department of Internal Medicine and Medical Therapeutics, University of Pavia Medical School, Pavia, Italy; ^5^ Cardiothoracic and Vascular Department, Unit of Respiratory Diseases, Istituto di Ricerca e Cura a Carattere Scientifico (IRCCS) Policlinico San Matteo, Pavia, Italy; ^6^ Department of Medical Sciences, Unit of Pneumology, Azienda Socio-sanitaria Territoriale (ASST) Crema, Crema, Italy; ^7^ Diagnostic Imaging and Radiotherapy Unit, Department of Clinical, Surgical, Diagnostic, and Pediatric Sciences, University of Pavia Medical School, Pavia, Italy; ^8^ Radiology Institute, Fondazione Istituto di Ricovero e Cura a carattere Scientifico (IRCCS) Policlinico San Matteo, Pavia, Italy; ^9^ Department of Surgical, Medical and Molecular Pathology and Critical Care Medicine, University of Pisa, Pisa, Italy

**Keywords:** interstitial lung diseases, idiopathic pulmonary fibrosis, lung cancer, predictors, gender

## Abstract

**Background:**

Interstitial lung diseases (ILDs) comprise a family of heterogeneous entities, primarily characterised by chronic scarring of the lung parenchyma. Among ILDs, idiopathic pulmonary fibrosis (IPF) is the most common idiopathic interstitial pneumonitis, associated with progressive functional decline leading to respiratory failure, a high symptom burden, and mortality. Notably, the incidence of lung cancer (LC) in patients already affected by ILDs—mainly IPF—is significantly higher than in the general population. Moreover, these cases are often neglected and deprived of active oncologic treatments.

**Methods:**

We here aim to identify variables predictive of outcome (mortality) in a multicentre retrospective cohort of ILD associated with lung cancer, collected from 2018 to the end of 2023. Overall, 73 cases were identified, and exhaustive clinicopathologic data were available for 55 patients. Among them, 42 had IPF. The entire dataset was then analysed by using the JMP partition algorithm (JMP-Statistical Discoveries, from SAS), which can choose the optimum splits from many possible trees, making it a powerful modelling and data discovery tool.

**Results:**

The average age at lung cancer diagnosis was 71.4 years, whereas the average age at IPF diagnosis was 69.5 years. The average Charlson Comorbidity Index was 4.6. Female patients constituted 28.3% (15) of the evaluated cases. The most frequent tumour histotype was adenocarcinoma (45.2%), and in more than 60% of the cases (67.9%), cancer was diagnosed at an early stage (TNM I–II–IIIA). A significant gender difference emerges regarding the overall patient survival, and quite unexpectedly, surgical approach to IPF-associated LC and the detection of serum autoantibodies are among the strongest outcome predictors.

**Conclusions:**

The analysis performed is descriptive and successfully identifies key features of this specific and rare cancer population. IPF-associated LC emerges as a unique malignant disease defined by specific gender and histopathologic clinical and molecular parameters, which might benefit from active treatments.

## 1 Introduction

Interstitial lung diseases (ILDs) are a heterogeneous group of lung diseases characterised by fibrosis (scarring) of the parenchyma. ILD classification is based on histopathological, radiologic, aetiologic, and clinical criteria (1–3). Idiopathic pulmonary fibrosis (IPF) is a rare, chronic, and progressive fibrosing interstitial pneumonia occurring mainly in the elderly population, with greater incidence after the age of 60 and characterised by a highly poor prognosis (4). Although the aetiology of this disease remains largely unexplored, IPF is the most common of ILD, and its incidence appears to be increasing (5, 6). IPF is characterised by irreversible loss of lung function due to fibrosis, manifesting as chronic cough and progressive worsening of dyspnoea, with a median survival from diagnosis of approximately 2–4 years. However, it is difficult to predict the clinical course due to the significant variability among cases. Lung cancer (LC) is one of the most significant comorbidities occurring in patients with ILDs, mainly IPF (7). It is well established that the two diseases share common risk factors (smoking habits) and some molecular traits (8–10). The combination of the two pathologies is a severe condition, with a negative impact on both mortality and the quality of life of patients (11, 12). It is therefore essential to structure a therapeutic strategy that improves survival prospects, as well as the quality of life of these patients (13). Unfortunately, no standardised diagnostic and therapeutic approaches have been defined for these neglected cases. Sometimes, a palliative approach is the only option. It is, thus, mandatory to identify parameters/variables that can help in patient stratification and outcome prediction to identify effective strategies for managing concomitant diseases. The rationale of the present work is to analyse demographic and clinical data from a cohort of IPF patients with concomitant lung cancer, aiming to rank the various risk factors and identify mortality predictive markers.

## 2 Patients and methods

### 2.1 Patient identification and selection

The study retrospectively analyses data from a consecutive series of patients who were observed at three reference ILD and thoracic oncology centres in Italy: the IRCCS San Matteo Hospital Foundation in Pavia, the Azienda Aspedaliera Specialistica dei Colli in Naples, and the Policlinico Riuniti in Foggia. Data collection spans from the beginning of 2018 to the end of 2023. This is not a clinical trial. Exhaustive clinical reports regarding the evaluated population are available in **Table 1**. Informed consent from each patient was routinely collected at hospital admission in accordance with standard hospital procedures. Patient data were collected through consultation of operating directories; oncological, pneumological, and pathological reports; and discharge letters.

**Table 1:** Demographic and clinical features of the evaluated population.

Variable	*n* (%)
Sex	
Male	40
Female	15
Average age at IPF diagnosis (years)	69
Average age at LC diagnosis (years)	71.4
Average interval between the two diagnoses (years)	2.7
CCI (average)	4.6
Cardiovascular comorbidities	35 (63.63)
Hypertension	20 (36.36)
Ischemic cardiopathy	11 (20)
Arrhythmias	4 (7.27)
Pulmonary comorbidities	14 (25.45)
COPD	11 (20)
PH	3 (5.45)
Gastrointestinal comorbidities	13 (26.63)
GERD	10 (18.18)
Achalasia	3 (5.45)
Cerebrovascular comorbidities	3 (5.45)
Stroke	2 (3.6)
Others	1 (1.8)
Endocrine comorbidities	26 (47.27)
Dyslipidemia	8 (14.54)
Disthyroidsm	6 (10.9)
Diabetes	8 (14.54)
Polymyalgia	4 (7.27)
Urinary tract comorbidities	9 (16.36)
IPB	4 (7.27)
CKD	5 (9.09)
Past oncologic comorbidities	7 (12.5)
Prostate cancer	5 (7.27)
Hodgkin lymphoma in remission	2 (3.63)
Smoking history	
Ex	28 (50, 90)
Current	11 (20)
Never	16 (29.09)
OT	21 (38, 18)
Autoantibodies	18 (32.72)
ILD subtype	
IPF	42 (73.36)
NSIP	5 (9.09)
Sarcoidosis	1 (1.81)
HP	1 (1.81)
CPFE	6 (1.90)
ILD therapy	
P	19 (34.54)
N	16 (29.09)
Mtx	6 (10.90)
Cts	3 (5.45)
None	10 (18, 18)
Interruption	3 (5.45)
Tumour histotype	
SCLC	5 (9.09)
NSC undiff.	3 (5.45)
SCC	11 (20)
ADC	36 (65.45)
PD-L1 average expression	19.9
0–10	27 (49.09)
11–49	16 (29.09)
50–100	12 (21.8)
Cancer stage	
1	24 (43, 63)
2	10 (18.18)
3	13 (23.63)
4	8 (14.54)
LC therapy	
Surgery	11 (20)
IC	4 (7.27)
CHT	20 (36.36)
RT	6 (10.90)
None	14 (25.45)
Average time to death (months)	
ILD acute exacerbation	8 (25)
Average BMI	29, 71
Average ECOG	1

### 2.2 Immunohistochemistry and genetics

Tumour samples were obtained from patients who underwent surgical resection or biopsy. The sample was considered eligible for the study if the tumour morphology was preserved and a minimum of 100 cancer cells were present in the tissue section. Morphologic classification was assigned according to the last World Health Organisation lung cancer classification criteria (14), and the primary site of origin was confirmed by the immunohistochemical phenotype (thyroid transcription factor-1, p40 (15, 16)) by the two senior lung pathologists. According to recent guidelines (17), formalin-fixed paraffin-embedded (FFPE) tumour samples were retrospectively selected and submitted to immunohistochemical assay or analysis of PD-L1 (programmed death ligand 1) expression levels using a laboratory-developed test designed to optimise the use of the anti-PD-L1 22C3 antibody (Dako) on the Omnis autostainer (Dako, AGILENT technologies, Santa Clara (CA), USA) with the Envision FLEX (Dako) revelation system. PD-L1 IHC using the PD-L1 IHC 22C3 pharmDx kit was performed according to manufacturer recommendations (website at http://www.ventana.com/ventana-pd-l1-sp263-assay-2/). The IHC protocol was performed on specimens sectioned at a thickness of 3 μm and stained on positively charged glass slides stored at 4°C within 3 days after sectioning, according to already published protocols (18). Tumour proportion score (TPS) was evaluated according to published guidelines (14). PD-L1 expression was classified into TPS < 1% (negative), TPS 2% to 5% (weak), TPS 6% to 49% (positive), and TPS ≥ 50% (strong) (19–21). To assess the tumour mutational profile, tumour DNA was extracted using the DNA FFPE tissue kit (Omega, Norcross, GA, USA) according to the guidebook, and concentrations were detected by Qubit^®^ 2.0 fluorometer dsDNA HS assay kit (Thermo Fisher Scientific Inc., Waltham, MA, USA). Genetic analysis was performed according to routine procedures and widely described protocols (22–24), based on Next-Generation Sequencing (NGS) technology targeting 94 genes and 284 single-nucleotide polymorphisms.

### 2.3 Statistical analysis

Statistical analysis was carried out using the Excel add-in package. The continuous variables were expressed as the mean value ± the standard deviation (SD), and the latter was compared using the Student’s *t*-test for independent variables. The nominal variables were compared using *χ* ^2^ tests. The Kaplan–Meier method was used to generate the cumulative survival curves, and the log-rank test was used to calculate the differences between the curves. A *p*-value < 0.05 was considered statistically significant. The entire dataset was then tested using the JMP partition algorithm (JMP-Statistical Discoveries, from SAS; website at www.jmp.com), which can search for all possible subdivisions of the best predictors of response/outcome and event probability distribution.

## 3 Results

### 3.1 Demographic and clinical annotation

**Figure 1:**
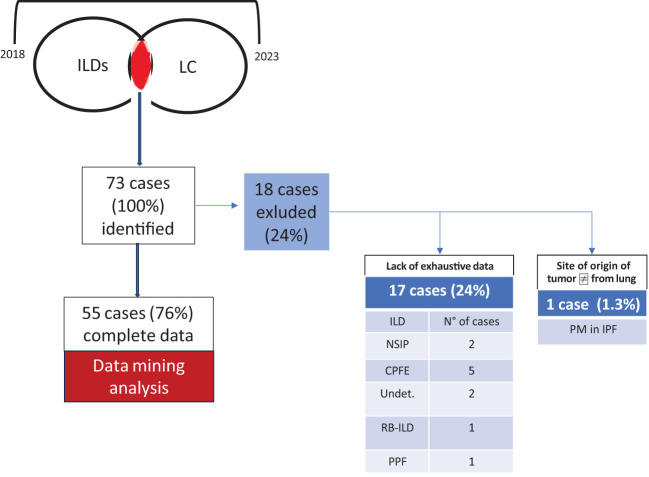
Study design. Overall, 73 cases were identified during the study interval (2018–2023); 18 of them were excluded due to a lack of exhaustive data and the absence of confirmed lung origin of the tumour. CTD, connective tissue disease-associated ILD; undet, undetermined fibrotic disease; RB-ILD, respiratory bronchiolitis interstitial lung disease; PM, pleural mesothelioma. One patient satisfied criteria for progressive pulmonary fibrosis (PPF) according to current guidelines. Serum autoantibodies detected in CTD patients included antinuclear antibodies (ANA) (all patients, 100%) and antibodies against topoisomerase 1 (Scl-70) (two patients, 33%). The patient with “undeterminate UIP” was treated with nintedanib due to a progressive fibrosing course. Patients with CTD-ILD received appropriate immunosuppressive treatment: methotrexate (50% of cases), mycophenolate (9%), and azathioprine (18%).

Lung cancer associated with ILDs is confirmed to be a rare condition, representing 2.1% of the total LC diagnoses in the three clinical centres during the study interval, whereas it aroused with an average interval of 2.7 years after the ILD diagnosis. Overall, the study screened and identified 73 patients; of them, 17 cases were excluded from the analysis due to the lack of complete clinicopathologic data, and one case was diagnosed with pleural mesothelioma. The remaining 55 ILD+LC patients were evaluated; their exhaustive features are reported in **Table 1**, while the study design is described in **Figure 1**. In the analysed population, most diagnoses (about 74% of cases) referred to IPF, while the remaining cases were diagnosis with nonspecific interstitial pneumonia (NSIP) and combined pulmonary fibrosis and emphysema (CFPE) in about 10% of cases. LC was associated with sarcoidosis in one case and hypersensitivity pneumonitis (HP) in another. The diagnosis of various ILDs was performed through the integration of anamnestic, clinicopathologic, radiological, and serological data, along with a multidisciplinary discussion, according to evidence-based diagnostic guidelines (25–27). An extended panel of autoantibody testing was applied to the blood of each case evaluated (28, 29). Autoantibodies were tested by immunoprecipitation, and all were IgG, except for the rheumatoid factor, which belongs to the IgM class by definition. Most ILD patients were treated with antifibrotic agents: 19 patients (35%) were treated with pirfenidone, 16 (29%) with nintedanib, six (11%) with methotrexate, and three (3.7%) with oral steroids. Male patients and smokers are at higher risk of developing both diseases (72.72% and 70.9% of cases, respectively). The pathogenic role of smoking is also coherent with the high incidence (63.63%) of cardiovascular illness in the analysed cohort. Similarly, 20% of patients had functional parameters of airway obstruction and/or static hyperinflation consistent with COPD. Respiratory function tests were performed periodically, with a focus on the analysis conducted at cancer diagnosis. Severe impairment of carbon monoxide diffusion was reported, with an average value of 50.6% of the expected (standard deviation [SD]: 17.8). For lung cancer detection, most cases (47, 85.45%) were diagnosed in stages I to IIIa LC, possibly reflecting the strict computed tomography follow-up for the ILD. A proliferative pattern of transformed cells was selectively limited to the fibrotic context. Indeed, in our cohort, 87% of cancer nodules were detected in the peripheral parenchyma in dense fibrotic areas. LC diagnosis was reached, in the vast majority of cases, through transthoracic CT-guided biopsy, and each case was evaluated by both interdisciplinary boards for thoracic neoplasms and for interstitial and rare lung diseases. Quite independently of the LC stage, patients’ overall survival after cancer diagnosis was 18.8 months, and 17.4 months in the IPF-only population (*p*-value 0.04). The expression of PD-L1 in tumour cells was extremely heterogeneous and overall positive, with an average of about 20%. With respect to cancer treatment decisions, they were based on interdisciplinary discussion, and it is not surprising that, due to the difficulty in managing these rare patients, treatment in some instances was not fully adherent to current lung cancer guidelines. Radiotherapy has generally been excluded to avoid further damage to the lung’s fibrotic parenchyma surrounding the tumour mass. Consequently, systemic cytotoxic chemotherapy was considered the best treatment choice, even in the early stages. On the other hand, the 25% of cases who received any treatment largely exceeds the 15% of those carrying advanced cancer diagnosis; this is mainly related to the worse performance status associated with concomitant respiratory failure. Overall, 10 patients were excluded from active anticancer therapy due to comorbidities and/or lung function severe impairment; three cases discontinued the initial treatment due to worsening performance status.

### 3.2 Outcome predictors

**Figure 2:**
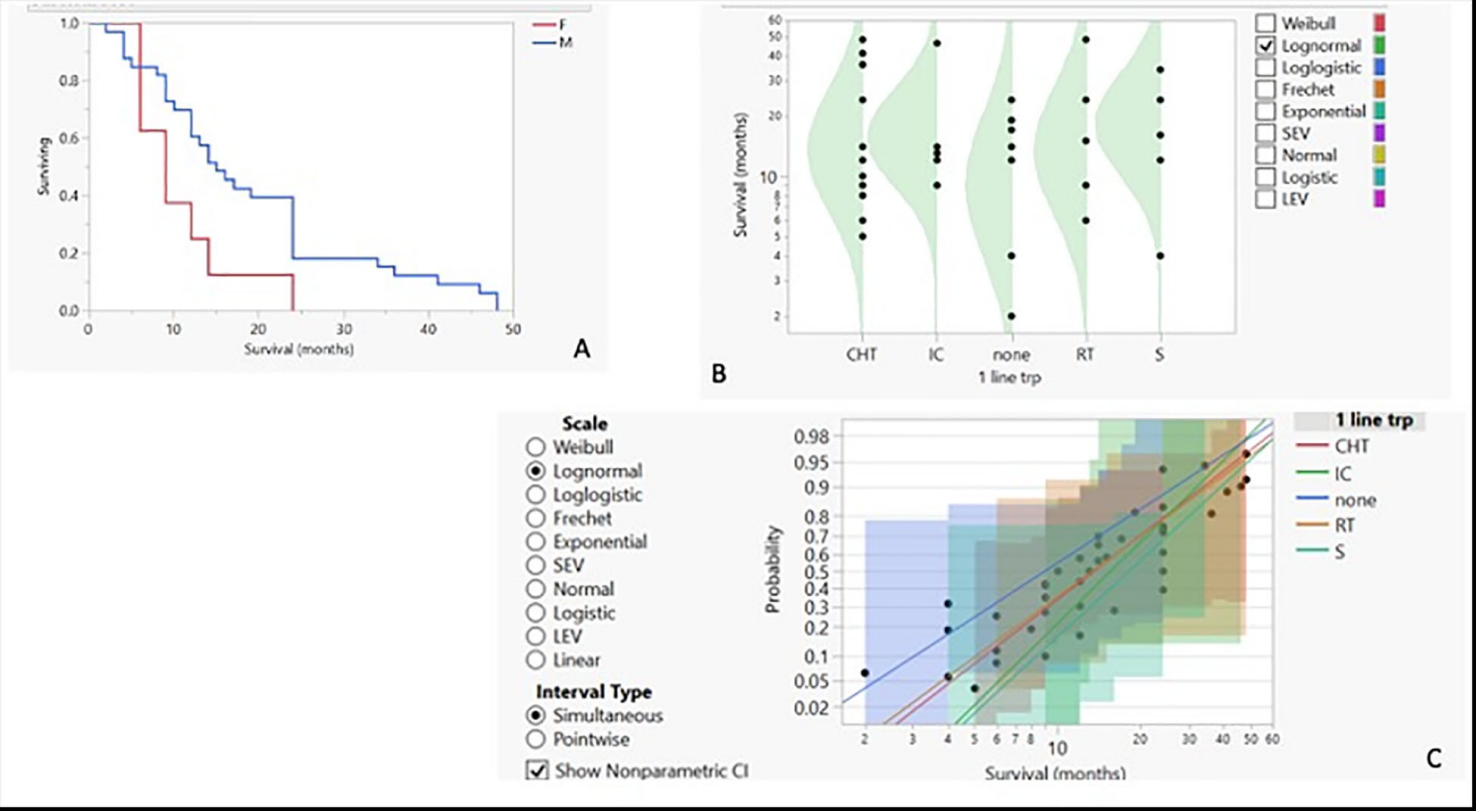
**(A)** Kaplan–Meier survival curves for IPF patients with LC based on their gender. Survival by first-line therapy against cancer: probability density curve **(B)** with logarithmic fit–nonparametric overlay **(C)**.

**Figure 3:**
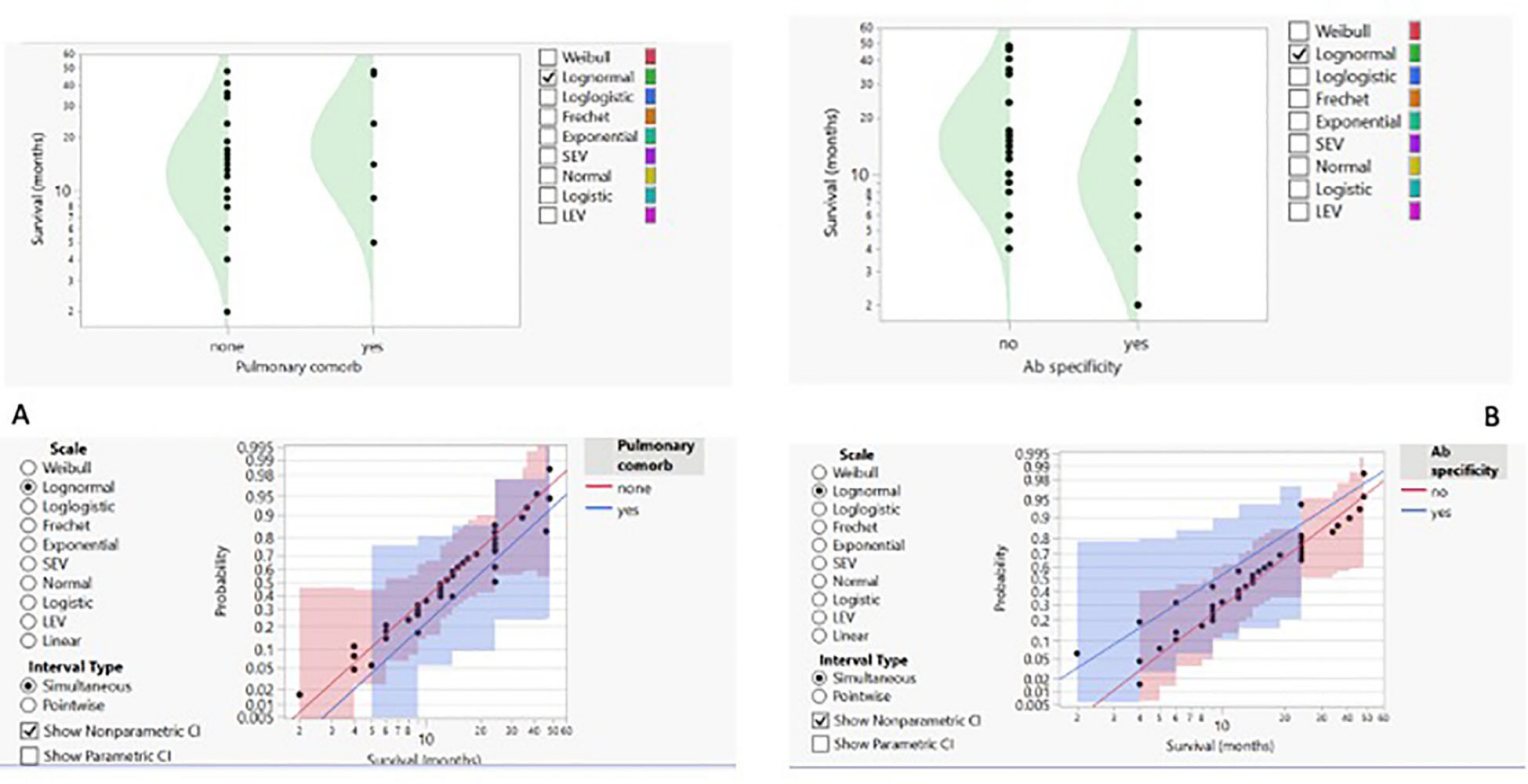
**(A)** Concomitant pulmonary comorbidity (COPD) and probability of survival shown as density curves with logarithmic fit–nonparametric overlay. **(B)** Detection of autoantibodies and probability of survival presented as density curves with logarithmic fit–nonparametric overlay.

We then proceeded to analyse the entire dataset with the aim of identifying the best predictors of patients’ outcomes. To obtain more reliable findings, the homogeneity of the dataset was increased by performing statistical analysis on the IPF cohort alone, the widest ILD population (42 out of 55 cases, 76.4%). Based on this, the row overall survival curve showed differences based on patients’ gender, with women having a lower survival rate than men (*p*-value < 0.05) (**Figure 2A**). Within the limits of the cohort in the study, the characteristics of female patients were similar to those of male patients, with the exception of a lower age at IPF diagnosis (61.25 years vs. 70.47 in the IPF cohort), and treatment options, as none of the women underwent surgery, regardless of clinical performance variables and cancer stage. On this evidence, we analysed IPF patients’ outcomes based on the therapy they received. The distribution of survival probabilities was analysed across the five groups representing the different first-line therapeutic strategies. This analysis highlights that the highest probability of survival occurs in the sub-population that underwent surgery for cancer (**Figures 2B, C**). With respect to the clinical variables that could impact patients’ survival, two conditions emerged as the most relevant. The first factor regards the co-existence of previous diagnoses of COPD, which, in the analysed cohort, acts as a protective factor and is associated with a higher survival probability (**Figure 3A**). Indeed, the overall survival of patients with associated obstructive conditions is 26 months. All except one of these patients were referred for chronic respiratory failure with the need for oxygen supplementation, and none of them underwent surgery. This point is of extreme interest since, although these patients were not referred for surgery, they were mainly treated with radiotherapy and chemotherapy. Notably, these tumours exhibited weak expression of PD-L1 (TPS% 22), suggesting that the immune reaction associated with COPD may have a protective effect. The lower number of CPFE cases does not allow for a proper comparison. However, within this limitation, patients with CPFE presented lower survival rates (13.3 months) in the absence of significant PD-L1 expression (TPS%: 20.7). The other clinical parameter independently associated with reduced survival is the presence of serum circulating autoantibodies (**Figure 3B**). None of the enrolled patients were referred for autoimmune disease diagnosis, nor was it confirmed after multidisciplinary revision.

### 3.3 Integrating molecular profiles into the clinical framework

**Figure 4:**
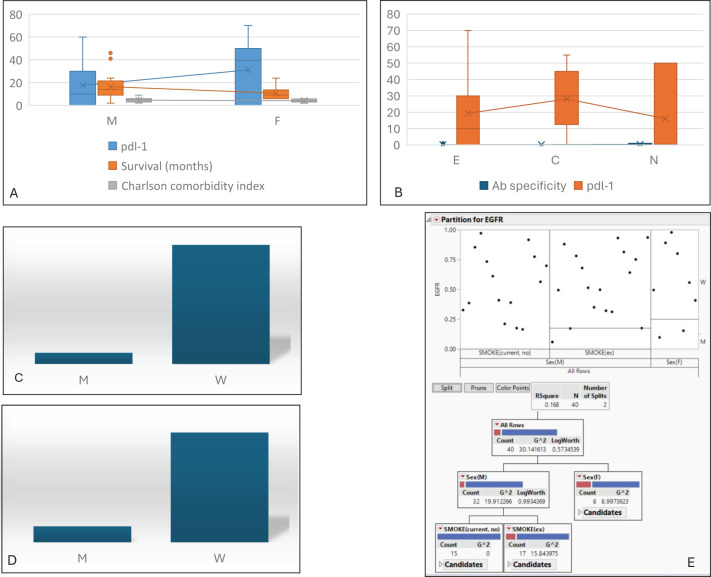
Molecular asset. **(A)** Distribution of PDL-1 level, survival, and CMI based on gender differences. **(B)** Distribution of autoantibody detection and PDL-1 levels based on smoking habits. Analysis of *EGFR* status vs. total amount of PDL-1 **(C)** and autoantibody detection **(D)**. **(E)** Partition analysis for *EGFR* status vs. gender and smoking, confirming that the wild-type receptor is more frequently associated with male sex and smoking habits. M, male; F, female; CMI, Charlson Comorbidity Index; E, past smoker; C, current smoker; N, never smoker; M, mutated; W, wild type.

With respect to IPF-associated cancer cases, we then proceed with a descriptive analysis not connected to outcomes and evaluate their molecular features to expand the knowledge base and better characterise malignant onset and progression. Recent evidence suggests a profibrotic role of the PD1/PD-L1 axis in IPF and mediastinal lymph nodes (30–32), paving the way for the therapeutic exploitation of low doses of immune checkpoint inhibitor (33, 34). These data strongly support the rationale for evaluating the expression of PD-L1 in IPF-associated cancer (**Figures 4A, B**). With respect to the cohort analysed, a key difference in PD-L1 tumour expression is related to patients’ gender, with significantly higher expression in women. Notably, this finding is not related to survival nor to autoantibody detection, and—quite unexpectedly—PD-L1 expression is uniformly distributed among current, past, and never smokers. We then investigated the impact of *EGFR* status and found that the wild-type receptor was associated with the highest PD-L1 levels (**Figure 4C**) and the presence of autoantibodies (**Figure 4D**). Moreover, when applied, the partition analysis for *EGFR* status (**Figure 4E**) confirmed that the wild-type status was more frequently detected in male and smoker subjects.

## 4 Discussion

The present study focuses on a retrospective analysis of a dataset regarding clinicopathologic and laboratory features of LC associated with ILD, collected from three reference centres in Italy. The design of the study itself limits mechanistic conclusions from the analyses; however, the statical approach used allows for the identification of previously unsuspected relationships between variables, highlighting critical pattern matches that could warrant future perspectives and experimental works. Thus, within this limitation, some considerations should be noted. The first is that, among ILDs, a selective preference for malignant growth in the IPF-related milieu can be recognised (35). Moreover, patients’ gender acts as an independent factor associated with overall survival. This trend seems to contrast with previously published data reporting no gender differences in survival in ILD patients with cancer (36), although a proper comparison between different studies should be limited by case heterogeneity. The significant difference in terms of lower survival in women with IPF and LC is an unexpected finding, not reported before in the literature. Men with IPF and LC, if subjected to surgery but in the absence of autoantibody detection, have a better prognosis. It is known that women have a greater predisposition to autoimmune disease (37–39), and this fact, in association with lower rates of surgical treatments compared to those in men (χ^2^: 5.17, *p*-value: 0.05 degrees of freedom), may be in some way related to lower survival. This observation allows us to conclude that, while being a woman with IPF is not associated with better survival, as already reported (40), women with concurrent IPF and LC had worse survival compared to male patients. Although limited, this result clearly suggests that surgical excision should be considered an effective option in early-stage LC in fibrotic patients—just as in nonfibrotic ones—and seems to be superior to radiotherapy in comparable populations. With respect to the cohort analysed, it should be underlined that women were rarely selected for surgery, and this issue should have had an impact on their survival. No clinical reasons emerged as causatively related to this observation, and these data support a rationale for a deeper investigation into the sociocultural role played by gender in the management of rare conditions such as IPF combined with LC. Interestingly, all except two patients who underwent surgery were treated with pirfenidone, which has already been reported to be associated with a lower risk of postoperative exacerbation (41, 42). Although the detection of autoantibodies in interstitial pneumonia with autoimmune features (IPAF) has been reported to be associated with better outcomes (43), more recent observations suggest that their presence in IPF should be associated with more rapid disease progression (44, 45). On the other hand, it should be noted that in scleroderma, the occurrence of specific autoantibodies (including anti-Scl70) has been strictly associated with a higher risk of cancer onset (46, 47). Thus, although autoantibody in IPF still requires full clarification, their detection in IPF associated with LC indicates poorer patient outcomes with potential implications. To further validate the study hypothesis, which is limited by the retrospective nature of the analysed data, we aim to produce exploratory findings that, after prospective validation, could help in the further development of a personalised approach to IPF-related LC. The results of this analysis highlight a gender-related expression of PD-L1 that is largely independent of smoking habits and more frequently associated with *EGFR* wild-type status. This observation allows us to hypothesise that immune checkpoint expression in this cancer type may be related to its activation in the surrounding fibrotic stroma rather than in response to cigarette smoke, as reported in primary lung cancer (48, 49). Thus, although genetic-driven tumour profiling is similar to that of IPF-free LC, as documented by *EGFR* behaviour and asset, the immune tumour status should be strictly related to the surrounding IPF stroma and the clinicopathologic features of the interstitial diseases. This preliminary observation suggests a strong rationale for further experimental research aimed at defining whether IPF-associated LC, once initiated, exploits the surrounding IPF traits as a selective force for progression.

## 5 Conclusions

Although limited, this study suggests that IPF is, among the ILDs, the one most strongly associated with LC onset.

Interestingly, our data are consistent with already published studies showing that autoantibodies are associated with worse IPF progression, according to mechanisms that involve the potential role of autoreactive B cells (see, for instance, refs (43, 44, 50).).

Although the role of autoantibodies in the pathogenesis of CTD-ILDs is better defined, recent data in the literature suggest an active role of B cells, plasma cells, and autoantibodies in IPF as well (51, 52). For example, in the study by Koether et al. (44), IPF patients exhibit an altered B-cell phenotype, and those with autoantibodies against the protein periplakin experience a rapid decline of lung function, suggesting a role for them in the progression of the disease. Tertiary lymphoid structures (sites of onset of humoral responses) have been found in the biopsies and explanted lungs of IPF patients, with a higher rate of activation of cells (assessed by the expression of CD40) in the advanced stages of the disease (53). The presence of prominent B-cell activation may contribute to the fibrotic remodeling process and suggest a potential role for anti-CD20 monoclonal antibodies in the treatment of IPF, similar to non-IPF ILDs. Both innate and adaptative immunity contribute to the fibrotic response characterising IPF. Although the role of T cells is better recognised in autoimmune ILDs or antigen-driven ILDs, growing evidence shows a role of T helper cells in IPF. Experimental models suggest a prominent role of Th2 and Th 17 subsets and their cytokines (54). In the context of excessive activation of T cells, a role may be played by a dysregulation of PD-1/PDL-1 axis, which has an inhibitory role on T-cell response. For example, in one study, PD-1 expression was found significantly downregulated in the T helper cells derived from the BAL of 20 patients with several ILDs, including IPF. This finding may explain the increased T-cell activity detected in these patients (55). Similarly, a population of T helper cells from peripheral blood mononuclear cells, with high expression of PD-1 and a cytotoxic phenotype, has been identified by mass cytometry in a population of SSc patients and has been associated with the presence and the severity of ILD. Overall, although of relevant clinical interest, our results cannot allow mechanistic conclusions/implications. Being a retrospective study, the total antibody levels could not be evaluated, nor could B-cell activation and soluble levels of PDL1. The findings of our study give a strong rationale for the development of perspective studies focusing on this topic, with potential gender-associated basis. Moreover, the results of the present work allow us to conclude that IPF-associated lung cancer can be defined as a separate malignant entity, carrying a definite clinicopathologic profile. This issue raises a strong rationale for the design of further studies aimed at evaluating the molecular asset of this cancer type. A second point that deserves deeper consideration is that, being a rare condition, IPF-associated LC should be managed by a multidisciplinary board with specific expertise and know-how in referral centers, as these patients could benefit, in some instances, from active and even aggressive (surgical) treatments.

## Data Availability

The datasets presented in this study can be found in online repositories. The names of the repository/repositories and accession number(s) can be found in the article/supplementary material.
